# Linking Social Anxiety to Depressive Symptoms in Adolescents: The Dual Mediating Roles of Perceived Social Support and Sleep Quality

**DOI:** 10.62641/aep.v54i2.2154

**Published:** 2026-04-15

**Authors:** Jing Gong, Qi Liu, Yajuan Ji, Jingsi Qu, Yaowei Xu, Xinchao Chen

**Affiliations:** ^1^Department of Psychiatry, Xiamen Xianyue Hospital, Xianyue Hospital Affiliated with Xiamen Medical College, Fujian Psychiatric Center, Fujian Clinical Research Center for Mental Disorders, 361012 Xiamen, Fujian, China; ^2^School of Clinical Medicine, Fujian Medical University, 350122 Fuzhou, Fujian, China

**Keywords:** adolescents, depressive disorder, social anxiety, perceived social support, sleep quality, mediating effect

## Abstract

**Objective:**

Social anxiety is a key risk factor for adolescent depression, yet its underlying mechanisms and subgroup differences remain unclear. This study explored the mediating roles of perceived social support and sleep quality in their link, and the moderating effects of visit type and gender.

**Methods:**

A retrospective observational study enrolled 386 depressed adolescents (12–18 years; 231 outpatients, 155 inpatients) from Xiamen Xianyue Hospital. Social anxiety, depressive symptoms, perceived social support and sleep quality were assessed using the Social Anxiety Scale for Adolescents, Self-Rating Depression Scale, Multidimensional Scale of Perceived Social Support and Pittsburgh Sleep Quality Index, respectively. Pearson’s correlation and Hayes’ PROCESS macro (Model 6) were applied for mediation/moderation analyses, with sensitivity testing via the Montgomery–Åsberg Depression Rating Scale (MADRS).

**Results:**

Inpatients and severe cases had higher Social Anxiety Scale for Adolescents (SAS-A), Zung Self-Rating Depression Scale (SDS) and Pittsburgh Sleep Quality Index (PSQI) and lower Multidimensional Scale of Perceived Social Support (MSPSS) scores (all *p* < 0.001); females had higher social anxiety (*p* = 0.003). Social anxiety correlated positively with depressive symptoms (r = 0.54, *p* < 0.001) and negatively with perceived social support (r = –0.49, *p* < 0.001). Mediation analysis showed a total effect of social anxiety on depressive symptoms (β = 0.304, *p* < 0.001), with direct effect (57.5%, β = 0.175) and total indirect effect (42.5%, β = 0.130). Key indirect pathways: ‘social anxiety → sleep quality → depressive symptoms’ (27.0%) and a serial mediation pathway via perceived social support and sleep quality (8.2%); perceived social support’s single mediation was marginally non-significant (*p* = 0.118). Moderation analyses revealed stronger direct effects in inpatients (β = 0.342 vs. 0.097, *p* < 0.001) and stronger sleep quality effects in females (β = 1.125 vs. 0.619, *p* = 0.006). MADRS sensitivity analyses confirmed consistency (path coefficient deviations <1%).

**Conclusions:**

Social anxiety affects adolescent depressive symptoms directly and via sleep-related mediation, moderated by visit type and gender. Targeting social anxiety and sleep quality may optimise precision prevention/treatment for adolescent depression.

## Introduction

Adolescence serves as a critical window for the rapid maturation of social skills and emotional regulation [1]. Depressive disorder during this stage is not only highly prevalent but also associated with significant functional impairment [2, 3]. A global systematic review and meta-analysis conducted by Racine *et al*. [4] reported high prevalence rates of depressive and anxiety symptoms in children and adolescents, along with an upward trend following the COVID-19 pandemic, underscoring the burden on this population. Additionally, analyses based on Global Burden of Disease data and longitudinal cohorts have highlighted the long-term effects of adolescent mental disorders on adult mental health [5]. Given the high prevalence and persistence of adverse outcomes associated with adolescent depression, elucidating its aetiological factors and modifiable mechanisms is of pressing clinical and public health importance [6].

Social anxiety, a disorder centred on fear and avoidance of social situations as its core features, is highly comorbid with depression in adolescents. Social anxiety has been repeatedly validated in school or community samples to predict or exacerbate depressive symptoms. However, most original studies rely on school or community cohorts. Whether their findings can be fully generalised to clinically referred adolescents, who differ substantially in disease severity, exposure to medications and hospitalisation and family/school support networks, remains to be verified [7]. Recent large-sample cross-sectional and longitudinal study further indicated that social anxiety not only correlates directly with low mood but may also indirectly amplify depression risk by altering individuals’ perceptions of social relationships and psychophysiological behavioural patterns such as sleep [8].

Systematic reviews have demonstrated that perceived social support exerts significant mediating or moderating effects in the anxiety–depression association [9, 10]. Moreover, the effects of different support sources (family, peers and significant others) on adolescents’ emotional outcomes are heterogeneous, highlighting the necessity of source-specific assessment in clinical samples [11]. Cohort studies suggest that sleep problems play a partial mediating role in the transition from anxiety to depression, supporting the integration of sleep quality as a key behavioural pathway in the social anxiety–depression transmission model [12, 13]. Based on social support theory, social anxiety may undermine individuals’ perception and utilisation of available social support, thereby reducing psychological resilience and increasing vulnerability to depressive symptoms [14]. In parallel, sleep regulation theory posits that persistent emotional hyperarousal associated with social anxiety can disrupt sleep homeostasis, and subsequent deterioration in sleep quality further impairs emotion regulation capacity and facilitates the development of depressive symptoms [13]. From a biological perspective, social anxiety is associated with activation of the hypothalamic–pituitary–adrenal (HPA) axis and heightened stress responses, which may disturb serotonin- and dopamine-related neurotransmitter systems involved in mood regulation [15]. In addition, social anxiety may indirectly exacerbate depressive symptoms by altering sleep–wake rhythms and modulating neural circuits related to the perception of social support [16]. Together, these psychosocial and neurobiological mechanisms provide a coherent physiological rationale for selecting perceived social support and sleep quality as mediating variables.

This study proposes the following hypothesised pathways: social anxiety may influence depressive symptoms directly and indirectly through two mechanisms: a single-mediator pathway (social anxiety → sleep quality → depressive symptoms) and a serial mediation pathway (social anxiety → perceived social support → sleep quality → depressive symptoms). With a focus on adolescents with depression receiving outpatient or inpatient psychiatric treatment, this study aimed to (1) examine the association between social anxiety and depressive symptoms, (2) evaluate the dual mediating roles of perceived social support and sleep quality and (3) compare path differences across gender and visit type (outpatient vs. inpatient) subgroups. By doing so, this study provides external validity for the proposed ‘psychological–behavioural serial pathway’ within clinical populations and identifies priority intervention targets in real-world psychiatric settings. The findings may inform evidence-based strategies for screening, treatment and follow-up of adolescent depression.

## Materials and Methods

### Study Design

A retrospective observational study design was adopted. Medical records of 386 adolescents (aged 12–18 years) diagnosed with depressive disorder were retrieved from the Department of Psychiatry at Xiamen Xianyue Hospital, including 231 outpatients and 155 inpatients. Historical clinical data of adolescent patients with depression treated in outpatient and inpatient settings at a tertiary psychiatric hospital were retrospectively reviewed, and statistical models were constructed accordingly.

The study was conducted between January 2024 and June 2025, adhering to the ethical principles of the Declaration of Helsinki. All study procedures were approved by the Medical Ethics Committee of Xiamen Xianyue Hospital (Approval No.: 2025-KY-119). The entire research process comprised three stages: participant recruitment and screening, standardised scale assessment, and data entry and statistical analysis. Standard operating procedures were implemented at each stage to ensure data quality and methodological consistency.

### Study Participants

#### Recruitment Method

Consecutive sampling was employed. Potential participants were screened by psychiatrists using the electronic medical record system of Xiamen Xianyue Hospital, covering outpatient and inpatient populations. The screening period extended from January 1, 2024, to June 30, 2025. Initially, relevant International Classification of Diseases, 10th Revision (ICD-10) diagnostic codes were queried to identify potentially eligible cases. Subsequently, two researchers independently reviewed the medical records to determine eligibility in accordance with the predefined inclusion and exclusion criteria. Any discrepancies were resolved through discussion until consensus was reached.

#### Inclusion and Exclusion Criteria

Inclusion criteria: (1) age between 12 and 18 years, calculated based on the date of birth recorded on the identification card (≥12 and ≤18 years at enrolment); (2) diagnosis of depressive disorder confirmed independently by two senior psychiatrists in accordance with the Diagnostic and Statistical Manual of Mental Disorders, Fifth Edition [17], supported by clear clinical documentation and standardised scale assessments; and (3) complete medical records, including sociodemographic characteristics, medical history, symptom descriptions and relevant psychometric assessment data required for this study. Given the retrospective study design and the use of anonymised clinical data, the requirement for written informed consent was waived by the Ethics Committee of Xiamen Xianyue Hospital.

Exclusion criteria: (1) comorbid schizophrenia spectrum disorders, autism spectrum disorder (Autism Diagnostic Observation Schedule-2 score ≥7) [17, 18] or severe intellectual disability (Wechsler Intelligence Scale IQ <70) [17, 19]; (2) presence of uncontrolled organic neurological conditions (e.g., status epilepticus or brain tumours) or severe physical illnesses (e.g., advanced heart failure); (3) history of substance or medication abuse within 1 week prior to diagnosis, indicated by a positive urine drug screening; and (4) incomplete medical records or missing key variables precluding reliable diagnosis or valid statistical analysis.

#### Sample Size Calculation

Sample size estimation was based on formulas for mediation analysis and pilot data, which indicated a correlation coefficient of *r* = 0.52 between social anxiety and depression and a medium mediation effect size (*f*^2^ = 0.18). Calculations were performed using G*Power (version 3.1, Heinrich-Heine-Universität Düsseldorf, Düsseldorf, Germany), with a two-tailed α level of 0.05 and statistical power (1–β) set at 0.90. Five covariates, including age and gender, were included in the model. The minimum required sample size was estimated to be 286 participants. Accounting for an anticipated attrition rate of approximately 20%, the target sample size was set at 350–400 participants, with approximately 60% outpatients and 40% inpatients.

### Measurement Tools

#### Depressive Symptoms

Depressive symptoms were assessed using the Chinese version of the Zung Self-Rating Depression Scale (SDS). The SDS consists of 20 items covering four domains, including affective and somatic symptoms, rated on a 4-point Likert scale. A total score ≥53 indicates the presence of depressive symptoms, with higher scores reflecting greater severity (mild: 53–62; moderate: 63–72; severe: ≥73) [20]. The Chinese version has demonstrated good internal consistency reliability (Cronbach’s α = 0.86) [21]. In the present study, Cronbach’s α was 0.63.

#### Depression Severity Assessment:

The clinical severity of depression was evaluated using the Montgomery–Åsberg Depression Rating Scale (MADRS) [22]. The MADRS comprises 10 items, including apparent sadness, reported sadness, inner tension, reduced sleep, reduced appetite, concentration difficulties, lassitude, inability to feel, pessimistic thoughts and suicidal ideation. Each item is scored from 0 to 6, with higher scores indicating greater severity. The total MADRS scores are categorised as none or very mild (0–6), mild (7–19), moderate (20–34) or severe (≥35) depression [23]. Ratings were independently performed by two trained psychiatrists. The Cronbach’s α for the MADRS in the present study was 0.65.

#### Social Anxiety

Social anxiety was assessed using the Chinese version of the Social Anxiety Scale for Adolescents (SAS-A) [14]. The scale consists of 18 items rated on a 5-point Likert scale ranging from 1 (‘not at all true’) to 5 (‘completely true’). The SAS-A comprises three subscales: fear of negative evaluation (FNE; eight items, score range of 8–40), social avoidance and distress in new situations or with unfamiliar peers (SAD-New; six items, score range of 6–30) and generalized social avoidance and distress in the presence of peers (SAD-General; four items, score range of 4–20). The total SAS-A score is obtained by summing the three subscales, yielding a range of 18–90, with higher scores indicating greater severity of social anxiety [24]. Previous studies have demonstrated that SAS-A is a reliable and valid instrument for assessing social anxiety in adolescents [25, 26]. In the current sample, the Cronbach’s α values were 0.82 for FNE, 0.80 for SAD-New, 0.81 for SAD-General and 0.82 for the total SAS-A.

#### Perceived Social Support

Perceived social support was assessed using the Chinese version of the Multidimensional Scale of Perceived Social Support (MSPSS) [27, 28]. The MSPSS consists of 12 items measuring support from family, friends and significant others, rated on a 7-point Likert scale (1 = strongly disagree to 7 = strongly agree). Total scores of 12–36, 37–60, and 61–84 indicate low, moderate and high levels of perceived social support, respectively [27]. The scale demonstrated excellent internal consistency (Cronbach’s α = 0.90), with α coefficients of 0.83, 0.85 and 0.88 for the subscales family, friends and significant others, respectively.

#### Sleep Quality

Sleep quality was evaluated using the Pittsburgh Sleep Quality Index (PSQI), which comprises 19 items generating seven component scores and a global score ranging from 0 to 21 [29]. A global PSQI score ≥8 indicates poor sleep quality [30]. The Chinese version of PSQI has shown good reliability in Chinese populations [31]. In the present study, the Cronbach’s α was 0.67.

### Study Implementation Process

#### Training and Quality Control

All research personnel completed a unified 8 h training programme prior to study initiation. The training covered standardised scale scoring criteria, procedures for clinical data extraction and ethical principles. Only investigators who achieved satisfactory performance (inter-rater reliability ≥0.85 for scale scoring) were permitted to participate. A standardised assessment manual was developed to clearly define operational procedures at each stage of the study.

#### Data Screening and Verification

Psychiatrists screened potential cases through the electronic medical record systems of outpatient and inpatient departments to verify eligibility in accordance with the predefined inclusion and exclusion criteria and the completeness of medical records and key variables. Subsequently, two researchers independently cross-checked the extracted medical records. A case was classified as complete only after confirmation that no critical information was missing.

#### Data Management

A dedicated database was established using EpiData (version 3.1, EpiData Association, Odense, Denmark), with predefined logical validation rules (e.g., age ranges and permissible scale score intervals). Data were independently entered by two research assistants, followed by cross-verification. Any discrepancies were resolved by referring to the original assessment forms. The database was encrypted and stored on the hospital research data platform, with access restricted to authorised personnel only. Missing data ≤5% were handled using multiple imputation, whereas samples with missing data >5% were excluded from the final analysis.

### Statistical Analysis

All statistical analyses were conducted using SPSS Statistics (version 26.0, IBM Corp., Armonk, NY, USA). Continuous variables were firstly tested for normality. Normally distributed variables are presented as mean ± standard deviation (SD), and they were compared between groups by using independent-sample *t* tests. Non-normally distributed variables are expressed as medians with interquartile ranges (Q_1_–Q_3_), and they were compared using Mann–Whitney *U* test. Categorical variables are presented as frequencies (percentages), and they were compared using chi-square (χ^2^) test. All analyses were two-tailed, with a significance level set at *p* < 0.05.

Mediation analyses were conducted using PROCESS macro (version 5.0; developed by Andrew F. Hayes, Distinguished Research Professor, Haskayne School of Business, University of Calgary, Calgary, Alberta, Canada). A serial mediation model (model 6) was specified, which allows two or more mediators to be entered in a predefined sequence and enables simultaneous testing of single and serial indirect effects. Social anxiety (SAS-A) was specified as the independent variable, depressive symptoms (SDS) as the dependent variable, and perceived social support (MSPSS) and sleep quality (PSQI) as sequential mediators. Gender, age and type of clinical setting (outpatient vs. inpatient) were included as covariates. Prior to formal mediation analysis, a multicollinearity test was performed for all variables involved in the model. Direct and indirect effects were estimated using a nonparametric bootstrap approach with 5000 resamples. Mediation effects were considered statistically significant when the 95% confidence interval (CI) did not include zero.

A moderation approach based on interaction terms was applied to examine whether path coefficients differed across subgroups defined by treatment setting and sex. Treatment setting (outpatient vs. inpatient) and sex (female vs. male) were specified as moderators (W). Within the same PROCESS framework, interaction terms (X × W; social anxiety × moderator) were entered into the model. A statistically significant interaction term (*p* < 0.05) was interpreted as evidence of a moderating effect, indicating significant differences in the corresponding path across subgroups.

Sensitivity analyses were performed by replacing SDS with the MADRS total score as the outcome variable to assess the robustness of the findings. The mediation analyses were repeated whilst maintaining the same model structure and covariate adjustments. Consistency in effect direction, magnitude and statistical significance was examined across models.

## Results

A total of 386 adolescents with depression were included in the final analysis, comprising 231 outpatients (59.84%) and 155 inpatients (40.16%). The sample included 177 males (45.85%) and 209 females (54.15%). Based on depression severity, 89 participants (23.06%) had mild depression, 223 (57.77%) had moderate depression and 74 (19.17%) had severe depression (Table 1).

**Table 1.** **Demographic characteristics, clinical features and core variable scores of the total sample and by clinical setting subgroup**.

Variables		Total (n = 386)	Inpatient (n = 155)	Outpatient (n = 231)	Statistic	*p*
Age, mean ± SD		15.18 ± 1.60	15.20 ± 1.67	15.17 ± 1.56	*t* = 0.20	0.844
Gender, n (%)					χ^2^ = 0.04	0.847
	Female	209 (54.15)	83 (53.55)	126 (54.55)		
	Male	177 (45.85)	72 (46.45)	105 (45.45)		
Disease duration in months, M (Q_1_, Q_3_)		8.95 (4.20, 18.65)	12.20 (6.30, 25.15)	6.50 (3.10, 12.60)	*Z* = −6.04	<0.001
Antidepressant use, n (%)					χ^2^ = 35.59	<0.001
	No	127 (32.90)	24 (15.48)	103 (44.59)		
	Yes	259 (67.10)	131 (84.52)	128 (55.41)		
SDS, mean ± SD		63.18 ± 9.60	64.97 ± 10.90	61.97 ± 8.43	*t* = 2.89	0.004
SAS-A, mean ± SD		66.84 ± 14.95	71.22 ± 12.92	63.91 ± 15.52	*t* = 4.84	<0.001
MSPSS, mean ± SD		45.94 ± 14.52	41.92 ± 13.73	48.63 ± 14.44	*t* = −4.57	<0.001
PSQI, mean ± SD		12.65 ± 4.74	14.38 ± 3.96	11.49 ± 4.87	*t* = 6.42	<0.001
Severity, n (%)					χ^2^ = 30.23	<0.001
	Light	89 (23.06)	25 (16.13)	64 (27.71)		
	Mid	223 (57.77)	80 (51.61)	143 (61.90)		
	Severe	74 (19.17)	50 (32.26)	24 (10.39)		

**Notes**: *t*, *t* test; *Z*, Mann–Whitney *U* test; χ^2^, chi-square test; SD, standard deviation; *M*, median; Q_1_, first quartile; Q_3_, third quartile; SDS, Zung Self-Rating Depression Scale; SAS-A, Social Anxiety Scale for Adolescents; MSPSS, Multidimensional Scale of Perceived Social Support; PSQI, Pittsburgh Sleep Quality Index.

### Basic Sample Characteristics and Inter-Group Comparison

Inpatients exhibited significantly higher scores on SDS, SAS-A and PSQI (all *p* < 0.01) than outpatients, whereas their MSPSS scores were significantly lower (*p* < 0.001). Inpatients also had a longer duration of illness (*p* < 0.001) and a significantly higher rate of antidepressant use (*p* < 0.001). No significant differences were observed between the two groups in terms of age or gender distribution (both *p* > 0.05, Table 1).

Female patients demonstrated significantly higher levels of social anxiety than male patients (SAS-A: 68.88 ± 15.25 vs. 64.43 ± 14.26, *p* = 0.003). No significant gender differences were observed for SDS, MSPSS, PSQI, duration of illness, antidepressant use, type of clinical setting or distribution of depression severity (all *p* > 0.05, Table 2).

**Table 2.** **Demographic characteristics, clinical features and core variable scores by gender subgroup**.

Variables		Female (n = 209)	Male (n = 177)	Statistic	*p*
Age, mean ± SD		15.27 ± 1.55	15.08 ± 1.66	*t* = 1.15	0.251
Disease duration in months, M (Q_1_, Q_3_)		9.20 (4.30, 19.80)	8.10 (4.00, 16.00)	*Z* = −0.74	0.456
Antidepressant use, n (%)				χ^2^ = 0.85	0.357
	No	73 (34.93)	54 (30.51)		
	Yes	136 (65.07)	123 (69.49)		
SDS, mean ± SD		63.63 ± 9.73	62.64 ± 9.44	*t* = 1.01	0.314
SAS-A, mean ± SD		68.88 ± 15.25	64.43 ± 14.26	*t* = 2.94	0.003
MSPSS, mean ± SD		46.11 ± 13.31	45.48 ± 12.76	*t* = 0.48	0.635
PSQI, mean ± SD		12.35 ± 4.60	11.99 ± 4.60	*t* = 0.76	0.446
Visit, n (%)				χ^2^ = 0.04	0.847
	Inpatient	83 (39.71)	72 (40.68)		
	Outpatient	126 (60.29)	105 (59.32)		
Severity, n (%)				χ^2^ = 0.0031	0.998
	Light	48 (22.97)	41 (23.16)		
	Mid	121 (57.89)	102 (57.63)		
	Severe	40 (19.14)	34 (19.21)		

Patients with severe depression had a significantly longer duration of illness (18.55 vs. 7.55 months, *p* < 0.001) and a markedly higher rate of antidepressant use (90.54% vs. 61.54%, *p* < 0.001) than those with mild to moderate depression. Patients with severe depression also exhibited significantly higher SDS, SAS-A and PSQI scores (all *p* < 0.001), alongside significantly lower MSPSS scores (*p* < 0.001). In addition, the proportion of inpatients was substantially higher amongst patients with severe depression (67.57% vs. 33.65%, *p* < 0.001). No significant differences were observed in age or gender distribution between the two severity groups (both *p* > 0.05, Table 3).

**Table 3.** **Demographic characteristics, clinical features and core variable scores by depression severity subgroup**.

Variables		Mild to moderate (n = 312)	Severe (n = 74)	Statistic	*p*
Age, mean ± SD		15.11 ± 1.60	15.49 ± 1.58	*t* = −1.87	0.063
Gender, n (%)				χ^2^ = 0.0069	0.986
	Female	169 (54.17)	40 (54.05)		
	Male	143 (45.83)	34 (45.95)		
Disease duration in months, M (Q_1_, Q_3_)		7.55 (3.48, 14.90)	18.55 (7.88, 26.87)	*Z* = −5.60	<0.001
Antidepressant use, n (%)				χ^2^ = 22.79	<0.001
	No	120 (38.46)	7 (9.46)		
	Yes	192 (61.54)	67 (90.54)		
SDS, mean ± SD		60.06 ± 7.61	76.33 ± 4.73	*t* = −23.29	<0.001
SAS-A, mean ± SD		63.77 ± 14.37	79.79 ± 9.40	*t* = −11.75	<0.001
MSPSS, mean ± SD		48.59 ± 11.56	34.18 ± 12.60	*t* = 9.47	<0.001
PSQI, mean ± SD		11.03 ± 4.07	17.02 ± 3.40	*t* = −11.72	<0.001
Visit, n (%)				χ^2^ = 28.63	<0.001
	Inpatient	105 (33.65)	50 (67.57)		
	Outpatient	207 (66.35)	24 (32.43)		

### Correlation Analysis of Core Variables

**Fig. 1.**
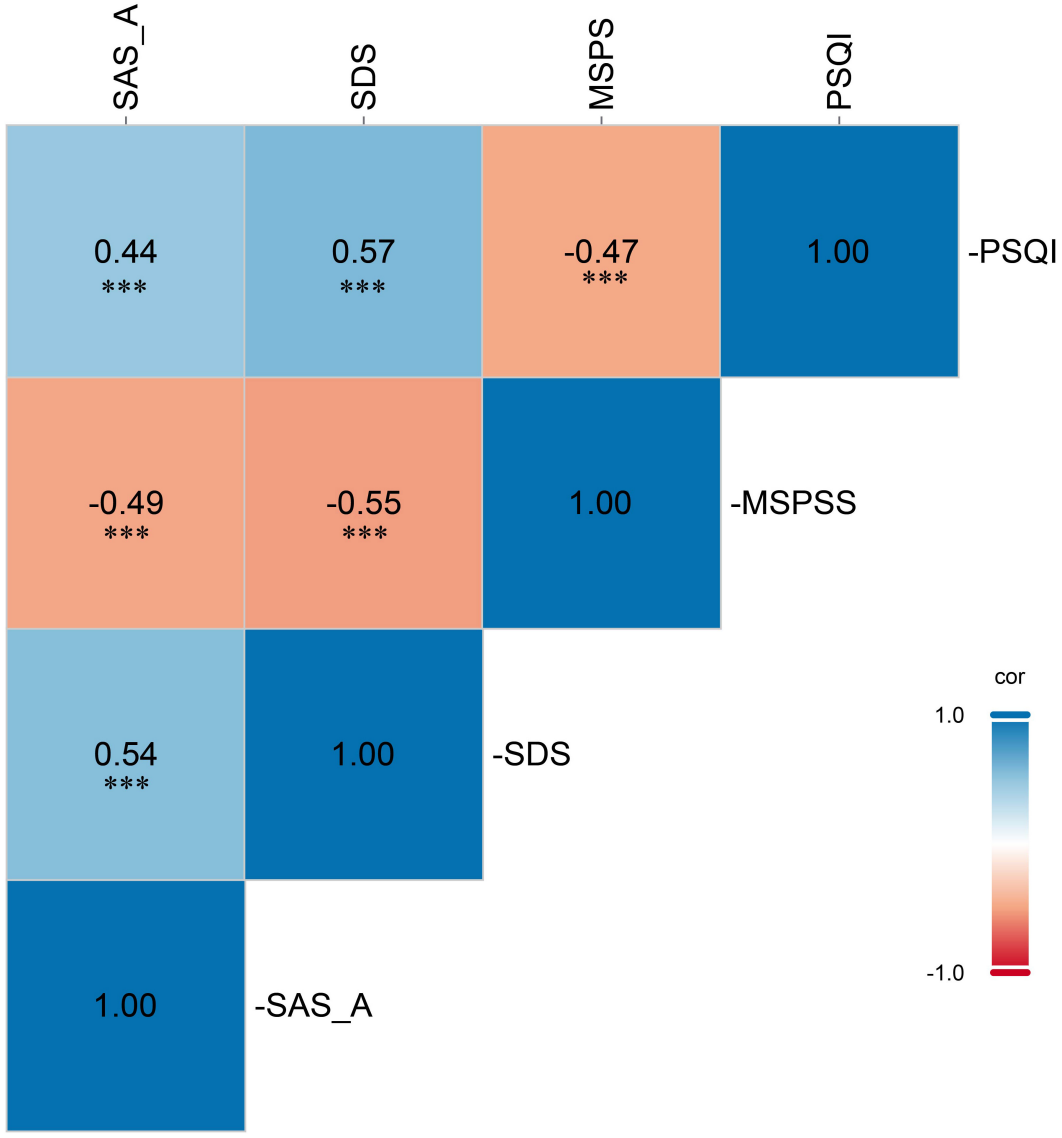
**Pearson’s correlations amongst social anxiety, depressive symptoms, perceived social support and sleep quality**. Abbreviations: SAS-A, Social Anxiety Scale for Adolescents; SDS, Zung Self-Rating Depression Scale; MSPSS, Multidimensional Scale of Perceived Social Support; PSQI, Pittsburgh Sleep Quality Index. *** *p* < 0.001.

Pearson correlation analyses (Fig. 1) demonstrated a strong positive association between social anxiety (SAS-A) and depressive symptoms (SDS, *r* = 0.54, *p* < 0.001). Social anxiety (SAS-A) was significantly negatively correlated with perceived social support (MSPSS; *r* = –0.49, *p* < 0.001) and positively correlated with poor sleep quality (PSQI; *r* = 0.44, *p* < 0.001). MSPSS showed significant negative correlations with PSQI (*r* = –0.47, *p* < 0.001) and SDS (*r* = –0.55, *p* < 0.001). In addition, PSQI was strongly and positively associated with SDS (*r* = 0.57, *p* < 0.001).

### Results of Mediating Effect Test

The mediating roles of perceived social support (MSPSS, M1) and sleep quality (PSQI, M2) in the association between social anxiety (SAS-A, X) and depressive symptoms (SDS, Y) were examined using Hayes’ PROCESS macro (model 6) and by controlling for gender, age and clinical setting. Assessment of multicollinearity indicated that the variance inflation factors (VIFs) for all variables ranged from 1.23 to 2.45. These values suggest the absence of problematic multicollinearity, indicating that the data were appropriate for mediation analysis. After covariates were adjusted, social anxiety significantly predicted perceived social support (R^2^ = 0.257, *F* = 32.86, *p* < 0.001). When social anxiety and perceived social support were entered simultaneously, the explanatory power for sleep quality increased further (R^2^ = 0.261, *F* = 26.86, *p* < 0.001). When social anxiety, perceived social support and sleep quality were jointly included, the model explained 39.5% of the variance in depressive symptoms (R^2^ = 0.395, *F* = 41.27, *p* < 0.001). The total-effect model of social anxiety predicting depressive symptoms was significant (R^2^ = 0.234, *F* = 29.03, *p* < 0.001, Table 4).

**Table 4.** **Robustness and goodness-of-fit indices of mediation models**.

Dependent variable	Independent variable(s)	R	R^2^	*F*	df	*p*
MSPSS (M1)	SAS-A + covariates (4)	0.507	0.257	32.86	4, 381	<0.001
PSQI (M2)	SAS-A + MSPSS + covariates (5)	0.511	0.261	26.86	5, 380	<0.001
SDS (Y)	SAS-A + MSPSS + PSQI + covariates (6)	0.629	0.395	41.27	6, 379	<0.001
SDS (Total Effect Model)	SAS-A + covariates (4)	0.483	0.234	29.03	4, 381	<0.001

As shown in Table 5, the total effect of social anxiety on depressive symptoms was significant (effect = 0.304, 95% CI: 0.245–0.364, *p* < 0.001). After the mediators were included, the direct effect remained significant (effect = 0.175, 95% CI: 0.113–0.236, *p* < 0.001), accounting for 57.5% of the total effect. The total indirect effect was 0.130 (95% CI: 0.087–0.179, *p* < 0.001), representing 42.5% of the total effect.

**Table 5.** **Decomposition of mediation effects and path-specific characteristics**.

Effect type	Path	Effect size	SE	95% Bootstrap CI (LLCI–ULCI)	*p*	% of total effect
Total effect	SAS-A → SDS	0.304	0.030	0.245–0.364	<0.001	100
Direct effect	SAS-A → SDS	0.175	0.031	0.113–0.236	<0.001	57.5
Indirect effect	SAS-A → MSPSS → SDS (Ind1)	0.022	0.015	−0.006–0.054	0.118	7.3
	SAS-A → PSQI → SDS (Ind2)	0.082	0.018	0.050–0.119	<0.001	27
	SAS-A → MSPSS → PSQI → SDS (Ind3)	0.026	0.008	0.011–0.042	<0.001	8.2
Total indirect effect	–	0.130	0.024	0.087–0.179	<0.001	42.5

**Fig. 2.**
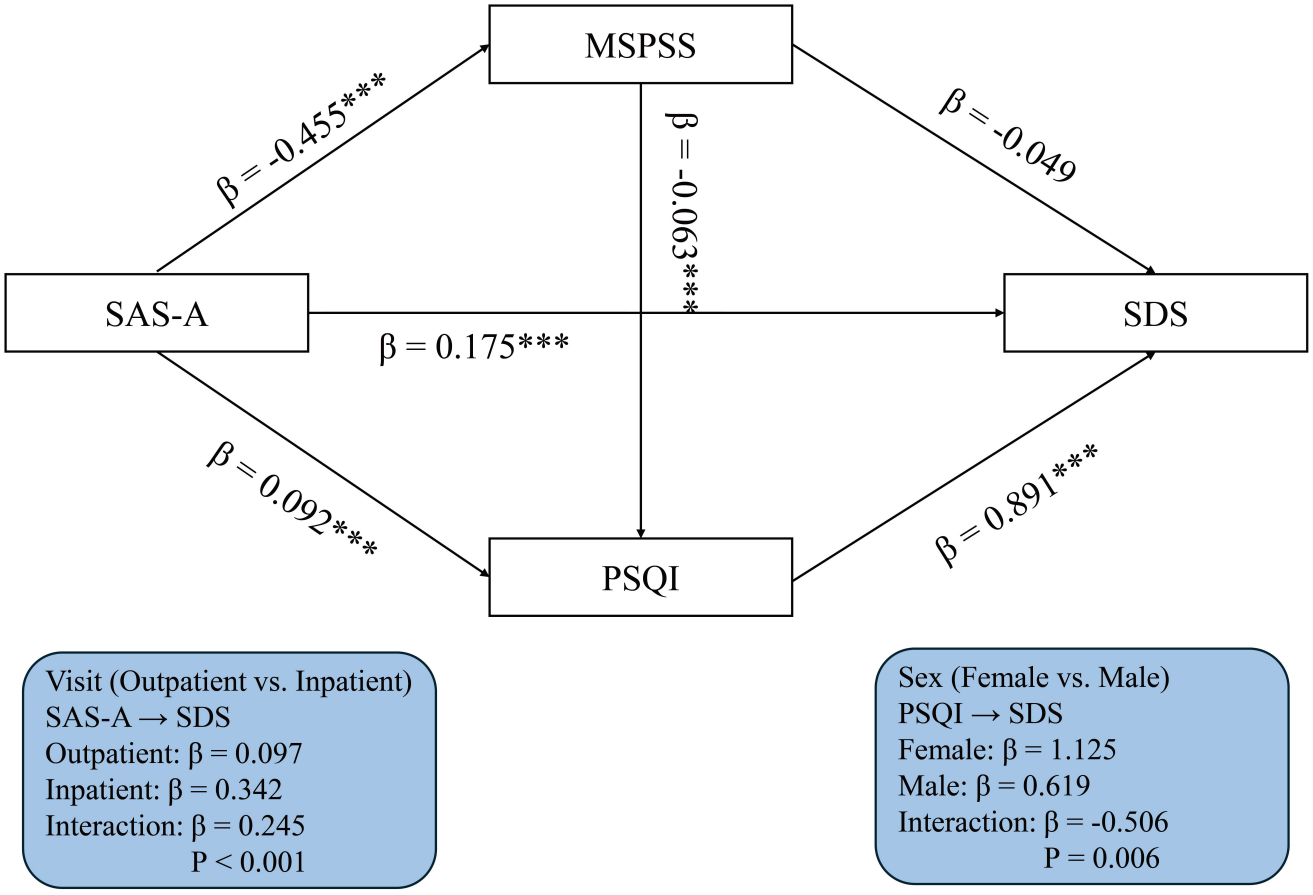
**Serial mediation model linking social anxiety to depressive symptoms via perceived social support and sleep quality**. Standardised coefficients are shown. Covariates included age, gender and visit type. *** *p* < 0.001.

Path-specific analyses indicated that the single mediation pathway ‘social anxiety → perceived social support → depressive symptoms’ was not statistically significant (Ind1, *p* = 0.118). By contrast, the pathway ‘social anxiety → sleep quality → depressive symptoms’ showed a significant mediation effect (Ind2, effect = 0.082, 95% CI: 0.050–0.119, *p* < 0.001), accounting for 27.0% of the total effect. Similarly, the serial mediation pathway ‘social anxiety → perceived social support → sleep quality → depressive symptoms’ was significant (Ind3, effect = 0.026, 95% CI: 0.011–0.042, *p* < 0.001), accounting for 8.2% of the total effect. The overall path structure and standardised regression coefficients are presented in Fig. 2.

### Subgroup Path Differences Based on Interaction Effects

An interaction-based moderation strategy (X × W) was applied to examine whether treatment setting and sex moderated specific paths within the mediation model. Interaction terms were entered into a single model, and the statistical significance of each interaction term was used to determine subgroup differences in key path coefficients (Table 6). In the analyses stratified by clinical setting (outpatient vs. inpatient), no significant group differences were observed for the effects of social anxiety on perceived social support or sleep quality nor for the effects of perceived social support or sleep quality on depressive symptoms (all interaction *p* values > 0.05). However, a significant difference emerged for the direct path from social anxiety to depressive symptoms, substantially stronger amongst inpatients than outpatients (β = 0.342 vs. 0.097; interaction β = 0.245, *t* = 3.88, *p* < 0.001).

**Table 6.** **Subgroup path differences based on interaction effects**.

Moderator	Path	Subgroup 1 (β)	Subgroup 2 (β)	Interaction (X × W) β	t	*p*
Type of visit (outpatient vs. inpatient)	SAS-A → MSPSS	−0.486	−0.385	0.101	1.06	0.292
	SAS-A → PSQI	0.137	0.086	−0.051	−1.62	0.107
	MSPSS → SDS	−0.036	−0.084	−0.048	−0.79	0.431
	PSQI → SDS	0.852	1.084	0.231	1.20	0.230
	SAS-A → SDS (direct effect)	0.097	0.342	0.245	3.88	<0.001
Gender (female vs. male)	SAS-A → MSPSS	−0.438	−0.477	−0.039	−0.44	0.657
	SAS-A → PSQI	0.113	0.132	0.020	0.67	0.505
	MSPSS → SDS	−0.014	−0.091	−0.077	−1.24	0.215
	PSQI → SDS	1.125	0.619	−0.506	−2.77	0.006
	SAS-A → SDS (direct effect)	0.123	0.238	0.116	1.84	0.066

In the gender-stratified analyses (female vs. male), no significant group differences were observed for the paths from social anxiety to perceived social support, from social anxiety to sleep quality nor from perceived social support to depressive symptoms (all interaction *p* values > 0.05). However, a significant gender difference was identified for the path from sleep quality to depressive symptoms, with a stronger effect in females than in males (β = 1.125 vs. 0.619; interaction β = –0.506, *t* = –2.77, *p* = 0.006). In addition, the direct effect of social anxiety on depressive symptoms was higher in males than in females, although this between-group difference reached marginal significance only (interaction *p* = 0.066).

Overall, clinical setting primarily moderated the direct effect of social anxiety on depressive symptoms, whereas gender mainly moderated the effect of sleep quality on depressive symptoms, indicating subgroup-specific differences in key mechanistic pathways.

### Sensitivity Analyses

All regression models demonstrated significant overall fit (all *p* < 0.001). Social anxiety (SAS-A) significantly predicted perceived social support (MSPSS), sleep quality (PSQI) and MADRS scores, with R^2^ values of 0.253 (*F*(4, 381) = 32.17), 0.258 (*F*(5, 380) = 26.34) and 0.412 (*F*(6, 379) = 43.89), respectively. Compared with the model in which SAS-A alone predicted MADRS (R^2^ = 0.246), the explanatory power increased by 67.5%, which was comparable to that observed in the SDS-based model (68.8%).

The total effect of social anxiety on the MADRS scores was significant (β = 0.318, SE = 0.032, 95% CI: 0.256–0.380, *p* < 0.001). The direct effect (β = 0.182, SE = 0.033, 95% CI: 0.117–0.247, *p* < 0.001) accounted for 57.2% of the total effect, whereas the total indirect effect (β = 0.136, SE = 0.025, 95% CI: 0.090–0.185, *p* < 0.001) accounted for 42.8%. The pattern of mediation effects was consistent with the primary analysis. The single mediation via sleep quality (SAS-A → PSQI → MADRS; β = 0.087, *p* < 0.001) and the serial mediation pathway (SAS-A → MSPSS → PSQI → MADRS; β = 0.028, *p* < 0.001) were significant, whereas the single mediation via perceived social support (SAS-A → MSPSS → MADRS; β = 0.021, *p* = 0.121) remained marginally significant. Deviations in the proportional contributions of each pathway relative to the SDS-based model were all below 1%, confirming the robustness of the findings.

## Discussion

This study focused on adolescents with depression receiving outpatient or inpatient psychiatric care. It systematically examined the association between social anxiety and depressive symptoms, the dual mediating roles of perceived social support and sleep quality and the moderating effect of clinical setting. The findings provide clinically grounded evidence for the behavioural–psychological mechanisms underlying social anxiety–depression comorbidity and offer empirical support for precision intervention strategies targeting adolescent depression.

The results showed that inpatients and adolescents with severe depression exhibited significantly increased social anxiety and sleep disturbance, markedly decreased perceived social support, lengthened illness duration and increased rates of pharmacological treatment. These observations are consistent with symptom profiles described by Van Meter *et al*. [32], who reported more severe affective symptoms and a higher burden of comorbid sleep problems amongst hospitalised adolescents with depression. Large-scale epidemiological studies and narrative reviews have indicated that female adolescents are more prone than males to anxiety-related symptoms, including social anxiety, likely due to gender-specific socialisation experiences and pubertal stress-response profiles [5, 6]. Notably, in the present study, 67.57% of adolescents with severe depression required hospitalisation, compared with 33.65% in the mild-to-moderate group. This finding suggests that imbalances within the ‘social anxiety–sleep–social support’ system may serve as early warning signals of depressive exacerbation, complementing community-based studies that often underrepresent clinically severe populations.

A robust positive association between social anxiety and depressive symptoms was observed in this clinical sample. Recent empirical studies and meta-analyses have consistently reported moderate correlations between social anxiety and depression in adolescents (r ≈ 0.3–0.6) [7, 8], supporting the stability of the correlation observed here (r = 0.54). Importantly, sleep quality showed the strongest correlation with depressive symptoms (r = 0.57), underscoring its central role in the underlying pathophysiology. This finding aligns with Baglioni and colleagues’ framework, which conceptualises sleep disturbance as an independent predictor of depressive onset [11]. Additionally, significant negative correlations were observed between perceived social support and social anxiety, sleep disturbance and depressive symptoms (r = –0.55 to –0.47), reinforcing the role of social support as a key protective factor in adolescent emotional health [12].

Mediation analyses indicated that the effect of social anxiety on depressive symptoms was primarily driven by a direct pathway, accounting for 57.5% of the total effect. This pattern is consistent with Hofmann’s theoretical model of ‘direct emotional activation’, wherein core social fears directly amplify depressive cognitive biases and negative self-appraisals [33]. Amongst the indirect effects, sleep quality emerged as the most prominent single mediator, accounting for 27.0% of the total effect. This finding echoes prior evidence showing that adolescents with social anxiety often experience circadian disruption due to social avoidance, and that sleep deprivation subsequently impairs emotion-regulation circuitry, including amygdala–prefrontal pathways, thereby amplifying depressive symptoms [34, 35, 36]. Notably, the single mediating effect of perceived social support was only marginally significant (*p* = 0.118), whereas its serial mediation through sleep quality was significant, accounting for 8.2% of the total effect. Guo *et al*. [37] reported that adolescents with higher social support exhibited better sleep quality, which, in turn, mitigated negative emotional outcomes. Similarly, Jin *et al*. [38] demonstrated a serial mediation pathway in which low social support increased depressive risk indirectly by promoting problematic internet use and worsening sleep quality. Collectively, these results suggest that the protective effect of social support on depression is largely realised via improvements in sleep. This may explain the nonsignificant independent effect of perceived social support observed in the present study.

In this clinical sample of adolescents seeking psychiatric care, perceived social support did not emerge as an independent mediator but exerted a significant effect through sleep quality. This pattern likely reflects a combined influence of true mechanism dependence and clinical sample characteristics. Patients in clinical settings tend to have a longer illness duration or greater symptom severity, accompanied by chronic stress exposure, dysregulation of the HPA axis and disrupted circadian rhythms. Such biological alterations may limit the capacity of subjectively perceived social support alone to immediately or directly restore impaired emotion regulation circuits [39, 40]. Consistent with the key characteristics of the present clinical adolescent sample, this finding may be further explained by the high proportion of severe depression (19.17%) and the persistence of comorbid social anxiety and depressive symptoms. These patients are likely to remain in a state of sustained physiological hyperarousal, with markedly disturbed sleep homeostasis (mean PSQI score = 12.65). Under such conditions, social support may firstly need to operate through modulation of biological rhythms and improvement of sleep quality, thereby facilitating the functional recovery of emotion regulation networks, such as amygdala–prefrontal circuits, before indirectly alleviating depressive symptoms [41]. In addition, adolescents with more severe clinical presentations often exhibit pronounced social withdrawal, which may impede the direct transmission and perception of social support. In this context, sleep represents a modifiable physiological pathway through which psychological resources can be translated into emotional improvement [42]. Together, this ‘psychological resources–physiological pathway–emotional outcome’ chain offers a novel perspective for understanding intervention challenges in adolescents with clinical depression. Clinically, the findings suggest that in adolescents with severe symptoms or those requiring hospitalisation, prioritising sleep-focused interventions whilst enhancing accessible social support may lead to more rapid emotional improvement than strategies aimed solely at increasing perceived support. However, longitudinal and experimental studies are needed to determine whether sleep functions as a necessary mediator in the translation of social support into improved emotional outcomes.

The subgroup differences in path coefficients were evaluated using an interaction-based approach (X × W) to examine whether treatment setting and sex moderated specific paths within the mediation model. Treatment setting and sex were selected as stratification variables on the basis of the study’s core hypotheses, considerations of multicollinearity and statistical power. Depression severity was strongly correlated with key study variables, and unequal subgroup sizes could have compromised the stability of stratified estimates. In the stratified analysis by treatment setting (outpatient vs. inpatient), no significant between-group differences were observed in the effects of social anxiety on perceived social support or sleep quality nor in the effects of perceived social support or sleep quality on depressive symptoms. The moderation effects identified through multigroup comparisons provide a rationale for precision-oriented interventions, with novelty arising from the delineation of pathway differences across the dual dimensions of clinical setting and sex. Moderation by treatment setting indicated that the direct effect of social anxiety on depressive symptoms was substantially stronger amongst inpatients (β = 0.342) than amongst outpatients (β = 0.097). This finding is consistent with prior evidence suggesting that hospitalisation contexts may amplify emotional burden through processes such as illness labelling and self-stigmatisation, which, in turn, undermine treatment engagement and prognosis [43, 44, 45]. Clinically, these results suggest that beyond routine medical management, inpatients may benefit from targeted interventions addressing social anxiety to mitigate its direct adverse effect on depressive symptoms. Such approaches could include cognitive–behavioural strategies focused on social avoidance, social skills training and exposure-based interventions, incorporated as integral components of inpatient rehabilitation and post-discharge follow-up.

Sex-specific moderation analyses further demonstrated that the association between sleep quality and depressive symptoms was stronger in females (β = 1.125) than in males (β = 0.619). This pattern aligns with findings reported by Hankin [46], and it may reflect a greater tendency amongst females to translate sleep disturbances into emotional distress via ruminative cognitive processes, whereas males may rely more on alternative emotion regulation strategies, such as behavioural diversion, which could attenuate this pathway. From a biological perspective, pubertal fluctuations in oestrogen and progesterone levels in adolescent females may heighten amygdala sensitivity to negative emotional stimuli whilst reducing prefrontal regulatory efficiency. As a result, emotional exhaustion following sleep deprivation may be more readily converted into depressive symptomatology [47, 48]. Collectively, these findings extend beyond simplified single-mediator frameworks by integrating core mechanisms with subgroup-specific differences. From a clinical standpoint, prioritising sleep assessment and intervention in female adolescents appears feasible and potentially impactful. Routine screening for sleep problems, early implementation of cognitive behavioural therapy for insomnia (CBT-I) or behavioural–circadian interventions and coordination with family- and school-based support may more effectively attenuate the amplification of depressive symptoms driven by sleep disturbance. By contrast, male adolescents may benefit from intervention packages that place greater emphasis on managing externalising behaviours and incorporating behavioural activation strategies, thereby complementing their distinct emotion regulation pathways.

In addition, a methodological explanation warrants consideration. The more pronounced indirect effect of perceived social support via sleep may partly reflect measurement overlap and shared variance, resulting in pathway ‘redistribution’ or suppression effects within the mediation model. In clinical samples, hospitalisation status, experiences of stigma and limited accessibility of social networks may attenuate the short-term buffering effect of subjectively perceived support, thereby shifting its influence toward an indirect pathway operating through sleep, a more proximal physiological mechanism of emotional regulation. Accordingly, clinical practice may benefit from a ‘parallel–phased’ strategy: prioritising the management of modifiable physiological or behavioural targets (e.g., sleep disturbances) in the short term whilst simultaneously initiating efforts to restore and strengthen usable social support resources. These efforts may include structured family involvement, school-based coordination and skills training to enhance the effective mobilisation of peer support, thereby facilitating the translation of psychological resources into emotional improvement.

Several limitations of this study should be interpreted with caution. Firstly, causal inference is constrained. The retrospective design permits identification of associations but precludes determination of temporal or causal ordering amongst variables, and the specific sequence of ‘social anxiety → sleep disturbance → worsening depressive symptoms’ cannot be conclusively established. Secondly, the internal consistency of certain key instruments (e.g., SDS and PSQI) was at the lower bound of acceptable reliability (Cronbach’s α = 0.63–0.67). Reduced measurement reliability may attenuate true associations between variables, leading to underestimation of mediation effect sizes. This limitation may also partially account for the marginal significance of perceived social support as an independent mediator (*p* = 0.118) because lower reliability could weaken its observed associations with social anxiety and depressive symptoms, thereby preventing the direct mediation pathway from reaching statistical significance. Thirdly, the single-centre design may limit generalisability, underscoring the need for multicentre studies incorporating adolescents from diverse geographic regions and socioeconomic backgrounds. Moreover, the clinical sample was drawn primarily from a tertiary psychiatric hospital where patients typically present with longer illness duration, greater symptom severity or more complex comorbidities. These characteristics differ substantially from those of adolescents with mild depression treated in community or primary care settings with respect to symptom burden, social support networks, access to healthcare resources and help-seeking behaviours. Consequently, the applicability of the findings to individuals with mild or early-stage depression should be interpreted cautiously. Future studies incorporating samples across multiple tiers of the healthcare system may enhance external validity. In addition, potential confounding factors, such as recent stressors and personality traits, were not assessed; subsequent research may address these limitations through structural equation modelling with more comprehensive covariate control. Finally, this study did not include an intervention component. Future work may build on the identified mechanism to develop integrated intervention strategies that prioritise sleep improvement whilst strengthening social support.

## Conclusion

The analysis of 386 adolescents with clinical depression found that hospitalised patients and with severe depression exhibited significantly increased levels of social anxiety and heightened sleep disturbances, accompanied with relatively decreased perceived social support. Female adolescents demonstrated higher social anxiety than males. Social anxiety was significantly associated with depressive symptoms, with the total effect predominantly driven by a direct pathway (57.5%), and additional indirect associations observed through a single mediation pathway via sleep quality (27.0%) and a serial mediation pathway via perceived social support and sleep quality (8.2%). The independent mediation effect of perceived social support was only marginally significant. Subgroup analyses indicated that clinical setting and sex moderated specific pathway strengths: the direct association between social anxiety and depressive symptoms was stronger in hospitalised patients, whereas the relationship between sleep quality and depressive symptoms was more pronounced in female adolescents. These findings were consistently supported by MADRS assessments, highlighting the relevant effect patterns of social anxiety, sleep quality and social support in clinical adolescent depression and their subgroup-specific variations. These results provide important insights into future longitudinal and intervention studies.

## Availability of Data and Materials

The experimental data used to support the findings of this study are available from the corresponding author upon request.
